# Elemental Distribution and Structural Characterization of GaN/InGaN Core-Shell Single Nanowires by Hard X-ray Synchrotron Nanoprobes

**DOI:** 10.3390/nano9050691

**Published:** 2019-05-03

**Authors:** Eleonora Secco, Heruy Taddese Mengistu, Jaime Segura-Ruíz, Gema Martínez-Criado, Alberto García-Cristóbal, Andrés Cantarero, Bartosz Foltynski, Hannes Behmenburg, Christoph Giesen, Michael Heuken, Núria Garro

**Affiliations:** 1 Institut de Ciència dels Materials (ICMUV), Universitat de València, 46980 Paterna (València), Spain; eleonora.secco@uv.es (E.S.); Heruy.Mengistu@uv.es (H.T.M.); alberto.garcia@uv.es (A.G.-C.); 2 ESRF—The European Synchrotron, 71 avenue des Martyrs, 38043 Grenoble, France; jaime.segura@esrf.fr (J.S.-R.); gema.martinez.criado@csic.es (G.M.-C.); 3 Instituto de Ciencia de Materiales de Madrid (ICMM), Consejo Superior de Investigaciones Científicas (CSIC), Sor Juana Inés de la Cruz 3, 28049 Madrid, Spain; 4 Institut de Ciència Maolecular (ICMOL), Universitat de València, 46980 Paterna (València), Spain; andres.cantarero@uv.es; 5 AIXTRON SE, Dornkaulstrasse 2, 52134 Herzogenrath, Germany; B.Foltynkski@aixtron.com (B.F.); H.Behmenburg@aixtron.com (H.B.); C.Giesen@aixtron.com (C.G.); M.Heuken@aixtron.com (M.H.)

**Keywords:** semiconductor nanowires, synchrotron probes, nano-scale resolution

## Abstract

Improvements in the spatial resolution of synchrotron-based X-ray probes have reached the nano-scale and they, nowadays, constitute a powerful platform for the study of semiconductor nanostructures and nanodevices that provides high sensitivity without destroying the material. Three complementary hard X-ray synchrotron techniques at the nanoscale have been applied to the study of individual nanowires (NWs) containing non-polar GaN/InGaN multi-quantum-wells. The trace elemental sensitivity of X-ray fluorescence allows one to determine the In concentration of the quantum wells and their inhomogeneities along the NW. It is also possible to rule out any contamination from the gold nanoparticle catalyst employed during the NW growth. X-ray diffraction and X-ray absorption near edge-structure probe long- and short-range order, respectively, and lead us to the conclusion that while the GaN core and barriers are fully relaxed, there is an induced strain in InGaN layers corresponding to a perfect lattice matching with the GaN core. The photoluminescence spectrum of non-polar InGaN quntum wells is affected by strain and the inhomogeneous alloy distribution but still exhibits a reasonable 20% relative internal quantum efficiency.

## 1. Introduction

Nanowire based GaN/InGaN heterostructures, such as quantum wells (QWs), are acknowledged as optimum support for highly efficient optoelectronic devices [1]. This is mostly based on two facts: first of all, the wide spectral range potentially covered by InGaN QWs, which suits perfectly the requirements of lighting and photovoltaic solar cells [2] and, on the other hand, the benefits of NW morphology in overcoming the main difficulties affecting planar geometry [3]. The latter include the drastic reduction of the compositional inhomogeneities and segregation affecting indium [4], the more effective strain release [5], and the possibility to grow heterostructures on the NW lateral surfaces (corresponding to non-polar *m*-planes), which expands the optically active area and increases its quantum efficiency [6]. The significant efforts dedicated to obtain GaN/InGaN core-shell NWs with high structural and optical quality have nowadays fructified with the demonstration of several NW-based devices, such as light emitting diodes [7,8], laser diodes [9], and photodiodes [10].

Since device performance relies on the composition, homogeneity, and quality of the heterostructures, a careful and complete characterization with focus on individual GaN/InGaN NWs is a crucial milestone in this field. Techniques that enable the study of single NWs with spatial resolutions ranging from the micro- to the nano-scale are required. Moreover, in nanoscale functional materials, it becomes very important to correlate structural and optical properties by applying different techniques to the same nano-object. Therefore, the use of non-destructive and contact-less techniques is mandatory. For a long time, the most employed technique for the study of the core-shell NWs had been high resolution scanning transmission electron microscopy (HR-STEM) [11,12,13]. The analysis of indium distribution and concentration in the QWs could be carried out by energy-dispersive X-ray spectroscopy (EDS) [14,15] with high spatial resolution but low detection limits at NW level. These measurements, however, tend to be time-costly; difficult; and, most importantly, imply the destruction of a part of the sample.

The nanometer scale spatial resolution achieved in recent years in third-generation synchrotrons enables the study of single NWs with complementary techniques, such as X-ray fluorescence (XRF), X-ray diffraction (XRD), and X-ray absorption near edge structure (XANES). These are key tools for the determination of the chemical composition and structural properties of materials in a non-destructive manner with high sensitivity and selectivity and, nowadays, with sufficient spatial resolution. Furthermore, these allow for the scanning the X-ray beam and performing maps of single NWs. These clear benefits have expanded the use of synchrotron X-ray nanoprobe to the investigation of single InGaN NWs [16,17,18,19], even to those including non-polar InGaN/GaN QWs [20,21,22].

This work aims to demonstrate the benefits of synchrotron-based X-ray spectroscopies for the study of GaN/InGaN core-shell NWs grown by catalyst-assisted metal-organic chemical vapor deposition (MOCVD) containing non-polar QWs on their lateral surfaces. These systems typically present inhomogeneities in their elemental composition, defect concentration, and strain fields at the nanoscale, and their characterization by conventional techniques has proved to be complex and costly. A hard X-ray synchrotron nanoprobe provides the multi-technique platform required for this kind of studies. The elemental composition and their distribution were characterized by XRF. Strain fields and crystal quality were probed by XRD. Local order effects and elemental segregation could be analysed by XANES spectroscopy. The information gathered by the combination of these complementary techniques can be then used to interpret the photoluminescence (PL) spectra of individual NWs. Finally, numerical simulations reinforce the consistency of the experimental results.

## 2. Materials and Methods

**Figure 1:**
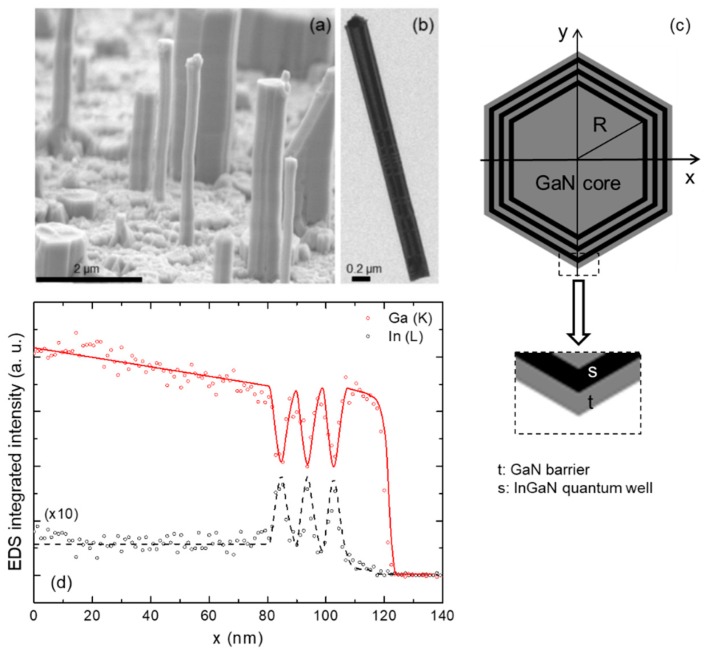
(**a**) SEM view of the as-grown nanowires (NWs) of the sample characterized in this study. (**b**) high resolution transmission electron microscopy (HR-TEM) of the single dispersed NW scanned by energy-dispersive X-ray spectroscopy (EDS). (**c**) Scheme of the cross-section of the core-shell GaN/InGaN NW and the magnification of its upper corner where *t* and *s* are the thickness of the InGaN quantum wells (QWs) and the GaN barriers, respectively. (**d**) Radial scans of the integrated intensities of the In L_α_ and Ga K_α_ peaks of the EDS spectrum taken at the top of the NW. The lines are the corresponding fitting curves.

Core-shell GaN/InGaN multiple-QWs (MQWs) on GaN NWs were grown by MOCVD in an AIXTRON 3 × 2” close-coupled showerhead reactor (AIXTRON SE, Herzogenrath, Germany). Details about the growth procedure are included in the supplementary information. A scanning electron microscopy (SEM) bird’s eye view micrograph of the sample is shown in Figure 1a, while a high resolution transmission electron microscopy (HR-TEM) image of a single dispersed NW is presented in Figure 1b. The NWs have diameters and heights ranging from 150 to 250 nm and 1.5 to 3 µm, respectively, and their longitudinal axis coincides with the *c*-axis of the wurtzite structure. Three GaN/InGaN MQWs form on the lateral non-polar *m*-planes of the NWs, whose expected structure is depicted in Figure 1c. In order to check whether the MQWs formed correctly during the growth process, energy-dispersive X-ray spectroscopy (EDS) measurements were performed along axial and radial directions on individual NWs. Figure 1d represents the integrated intensity of the In L_α_ and Ga K_α_ fluorescence lines recorded at each point of the scan along the radial direction of a NW. The core-shell MQWs and the barriers appear as regular oscillations in the In and Ga fluorescence signal. Linear EDS scans were also performed along the axial direction of the NW (see supplementary Figure S1), which indicates that no polar QWs formed on the top NW surface. By taking advantage of the 4 nm spatial resolution of EDS, we estimate the widths of the wells and the barriers by fitting the spectra of the lateral scans to the structure depicted in Figure 1c. This results in the following values: *R* = 93 ± 1.3 nm, *t* = 5.7 ± 3.2 nm, and *s* = 4.9 ± 2.3 nm. With these dimensions, the core represents 57% of the NW volume, while the shell takes the remaining 43%. On the other hand, quantifying the In concentration of the non-polar QWs is dissuasive due to the low signal to noise ratio of the EDS signal.

Individual NWs were dispersed on 200 nm-thick SiN windows for XRF, XRD, and XANES measurements at the nanoimaging station ID22NI (currently ID16B) of the European Synchrotron Radiation Facility (ESRF, Grenoble, France) with a multitechnique setup similar to the one described by J. Segura-Ruiz el al. [19]. The pink X-ray beam (∆E/E ~ 10^−2^) was focused providing a spot of 120 × 97 nm^2^ at an energy of 29.6 keV. The X-ray beam impinged nearly perpendicular to the sample surface and the XRF signal was detected at 15° using a single element energy dispersive silicon drift detector. XRF spectra were analysed using the program PYMCA [23]. XRD measurements were carried out with a monochromatic beam ((∆E/E ~ 10^−4^), with a spot size of 154 × 136 nm^2^ and an energy of 28.029 keV. The XRD signal was measured using a large field of view (94 × 94 mm^2^) fast readout low noise (FReLoN) charge coupled device (CCD) detector F-K4320T equipped with 3.3/1 demagnifying fibre optics taper (ESRF, Grenbole, France). The XRD data were analysed using both the Fit2D package [24] and the PYMCA program. Fit2D allows one to calibrate standard diffraction patterns, using the experimental parameters derived from the measurement of an Al_2_O_3_ reference sample. XANES spectra were recorded in XRF mode with a step size of 1 eV (equaling the resolution of the Si (111) double crystal monochromator) and an integration time of 1 s/point. The data were normalised and analysed with the IFFEFIT package [25].

Raman scattering and photoluminescence (PL) measurements were obtained using an optical microscope coupled to a T64000 triple spectrometer (Horiba Jobin-Yvon^®^, France) and a nitrogen-cooled CCD. The spectral resolution of the whole system was of 1 cm^−1^ at 500 nm. Microscope objectives of 100 and 40 magnification provided laser spot diameters of 1 and 4 µm for visible and UV excitation, respectively. The Raman spectra were collected in back-scattering configuration at room temperature with the 514 nm line of an Ar laser. PL measurements were performed at controlled temperature (7–300 K) with a 325 nm He-Cd laser.

## 3. Results

### 3.1. Composition and Structural Properties of Individual NWs by Raman Scattering

**Figure 2:**
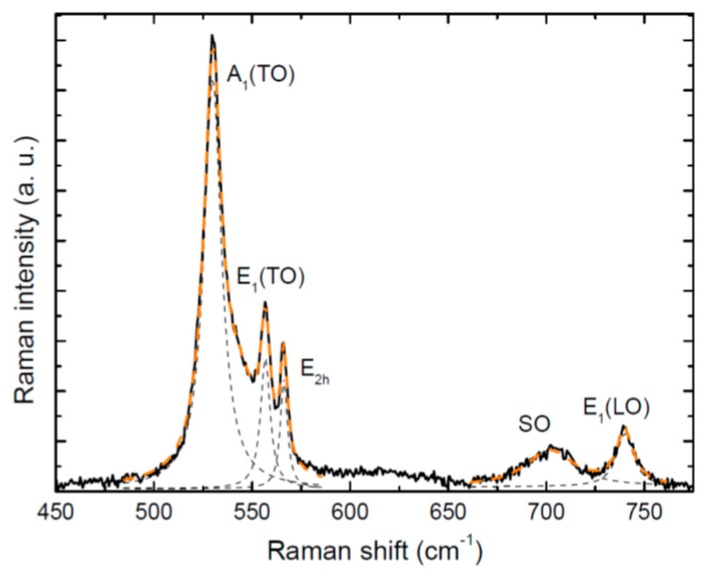
Representative unpolarized Raman spectrum of a single NW with its best fit (dashed lines).

Raman scattering can provide a first insight into In concentration and structural quality of the NWs. Micro-Raman measurements, with a spatial resolution of 1 µm, probe individual GaN/InGaN core-shell NWs that have been previously dispersed on an inert substrate. A representative Raman spectrum is shown in Figure 2. The observed Raman peaks have been fitted by Gaussians; their values match those of wurtzite strain-free GaN and should come mostly from the NW core. Due to the oblique facets of the NW, Raman selection rules are relaxed and four modes attributed to wurtzite GaN are observed: A_1_ (TO), E_1_ (TO), E_2h_, and E_1_ (LO). The FWHMs of the E_2h_ mode measured on several NWs are approximately 3.5 cm^−1^, indicating the good structural quality of the NWs. An additional peak centered around 701 cm^−1^ could match the frequency of the optical mode of the InGaN QWs. We cannot disregard, however, that this mode could correspond to a surface optical mode (SO), which typically appears between the TO and LO frequencies and is significantly intense in NWs due to the large surface-to-volume ratio [26]. Thus, no definitive conclusions are drawn regarding the In concentration and strain of the QWs from Raman investigations.

### 3.2. Elemental Distribution and In Concentration along Individual NWs by X-ray Fluorescence

**Figure 3:**
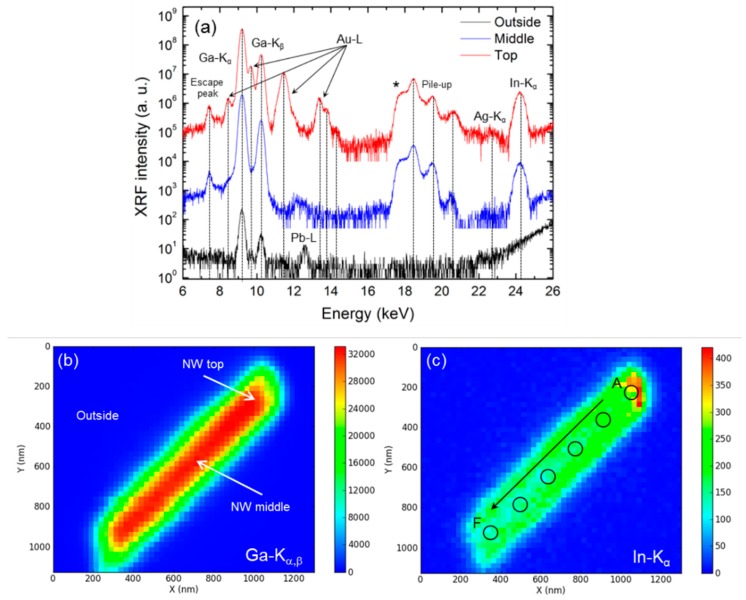
(**a**) Averaged X-ray flouresence (XRF) spectra from locations at the top (red) and middle (blue) of the NW, and at an outside region (black). The labels indicate the elements associated with each peak identified with PYMCA. The asterisk represents an artefact coming from the measurements. The XRF intensity color maps of Ga and In in the scanned area are shown in (**b**) and (**c**), respectively. Red (blue) color corresponds to high (low) fluorescence intensity (scale in counts). The black circles in (**c**) indicate the regions of the NW along its axis in which the In concentration has been estimated.

The composition of several GaN/InGaN core-shell NWs dispersed on a SiN thin window has been investigated recording XRF maps of the area enclosing the NW and its surrounding region. Figure 3a shows the XRF spectra measured at different regions of an individual NW: one at the top and at the middle of the NW, and at a location corresponding to the SiN window. Each spectra is obtained by averaging over 10 pixels (each pixel is 25 × 25 nm^2^) in the different regions of the map. The most intense peaks in the spectra are those corresponding to In, Ga, Au, and Ag. Outside the NW, Ga and In XRF intensities have vanished almost completely and an additional peak attributed to Pb is observed. The presence of weak Ga spectral peaks outside the NW can be justified by the long decaying tails (vertical and horizontal) of the non-circular X-ray beam and the low detection limits of this technique. Having a Pb signal is not surprising either, and it comes from the shielding system around the sample. Au and Ag were detected only at the top of the NWs and are attributed to the catalyst used for the NW growth. Ag, which can be present in very low levels in Au, has an estimated concentration of around 0.02% as calculated by PYMCA. This figure confirms the trace chemical sensitivity of synchrotron nano-XRF.

The distribution of Ga and In in the NW can be elucidated from the XRF intensity maps shown in Figure 3b,c. Figure 3b evidences that Ga is homogeneously distributed along the NW axis. On the other hand, the intensity of In increases from the bottom to the top end of the NW, as it is depicted in Figure 3c. The higher intensity of In at the top of the NW does not correspond to the formation of polar MQWs, as previously demonstrated by EDS. Au and Ag maps (see Figure S2) lead to the conclusion that no catalyst atoms are disseminated in the NWs. Further insight is achieved from longitudinal and radial scans of the XRF peak intensities (see Figure S3). These profiles confirm the trends that were previously found in the XRF maps: there is an InGaN shell structure with an increasing concentration of In towards the top of the NW. Ga atoms, on the other hand, are more evenly distributed. 

In order to estimate the In concentration in the core-shell MQWs, the relation between the intensity of the XRF emission of an element, *I_i_*, and its concentration, *C_i_*, are extracted from the maps using PYMCA software. In the case of thin samples, where enhancement effects due to additional excitation of the element of interest by the characteristic radiation of other elements can be neglected, these are related by the following equation:

(1) $$I_{i}\approx \;I_{0}C_{i}k_{i}d$$

where *I_0_* is the intensity of the incoming beam, *d* is the sample thickness, and *k_i_* accounts for the fluorescence yield, solid angle, and detection efficiency. The validity of Equation (1) for InGaN NWs has been extensively discussed by Gomez-Gomez et al. [17]. In a second step, the In concentration of the core-shell MQWs was estimated using the values for the thickness of the wells, barriers, and NW core obtained from the EDS analysis. Finally, the In concentration of the MQW on several points along the *c*-axis of the NW can be estimated. The obtained values are reported in Table 1 and correspond to the regions depicted in Figure 3c and are labelled from A to F. The error values are the result of the propagation of the uncertainties of the In concentration (given by PYMCA around 5%) and the well thickness (±2.7 nm). PYMCA errors are given mostly by the parameters of renormalisation used in the program; the dispersion of the XRF intensity (see Figure 3c) only contributes 0.13% and can be neglected.

### 3.3. Structural Properties of Individual NWs

**Figure 4:**
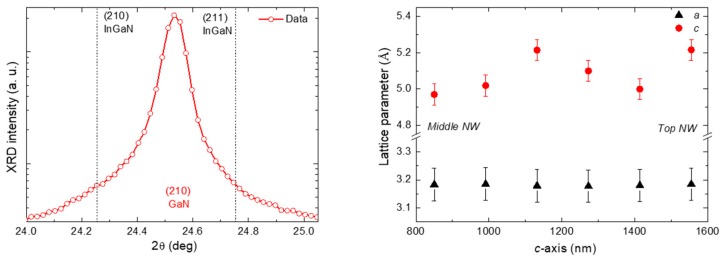
(**a**) XRD diffraction peak measured in the middle region of an individual NW and plotted in log-scale. The dashed lines indicate the positions of XRD peaks corresponding to unstrained GaN (210) and InGaN (210) and (211) with an average In concentration of *x* = 0.076. (**b**) Evolution of the wurtzite lattice parameters *a* and *c* along the z-axis of the NW, starting from the middle of the NW towards its top.

Nano-XRD measurements can address the crystal phase and the lattice parameters of different regions across single NWs. The short distance separating the CCD and the sample allowed that three diffraction peaks were measured simultaneously. The identification of these peaks, performed with Fit2D and PYMCA programs, pointed out that they correspond to (104), (210), and (211) reflections of unstrained wurtzite GaN (we are using 3 index notation (*hkl*), which is equivalent to (*hkil*) with *i* = −(*h + k*)). One of these Bragg reflections is shown in log-scale in Figure 4a. The value of the GaN wurtzite reflections expected in this case were calculated with PowderCell program [27]. Neither additional reflections nor asymmetries are observed (similar results are obtained for reflections (104) and (211)). Therefore, only diffraction peaks corresponding to unstrained GaN reflections were detected studying the XRD signal of the whole NW. This fact suggests that the core-shell InGaN MQWs are completely lattice matched to the strain-free GaN core. Since the In concentration has been measured by XRF, Vergard’s law can be used to calculate the lattice parameters corresponding to unstrained InGaN QWs, and from those the angular positions of the (210) and (211) Bragg reflections of InGaN. For an In concentration of 7.6%, XRD peaks should appear at 2θ_210_ = 24.262° and 2θ_211_ = 24.757°, which are depicted as dashed lines in Figure 4b. No distinctive XRD peak coming from the core-shell MQWs has been observed in the expected region, confirming that the core-shell MQWs are completely matched with the GaN core and therefore completely strained (compressed along *a* and *c* directions and expanded along *m*-direction).

The XRD reflexions allow one to calculate the interplanar distance *d_hkl_* using the the general relation between the interplanar distances (*d*), the Miller indices (*hkl*), and lattice parameters (*a* and *c*) in the wurtzite structure,

(2) dhkl=[43a2(h2+hk+k2)+l2c2]−12

and from them the lattice parameters *a* and *c* of the studied NWs can be measured. Symmetric reflections, such as (210), allow one to extract the lattice parameter *a* directly from Equation (2) and then use it to calculate the parameter *c* from non-symmetric reflections, such as (211). Scanning the X-ray beam along the NW axis, we can monitor any variation of the lattice parameters. Within the experimental errors, Figure 4b shows no evolution in *a* and a non-monotonous increase in *c* towards the NW top. The studied area starts at middle until the top of the NW. No signal was registered between the bottom and the middle of the NW due to the loss of the Bragg condition at imperfections on the NW. The maximum recorded variation of the axial lattice parameter *c* is 4%, which matches the increase in the In content of the NWs towards their top (see Table 1). The error associated with the lattice parameters is an average error, and it has been obtained from the difference of the value of the *c* parameter calculated from the reflections (211) and (104). The error of the calibration is the main contribution to the error of the diffraction angles, while the error of the fit performed by Fit2D program is three order of magnitude smaller than the calibration error and has not been taken into account.

**Figure 5:**
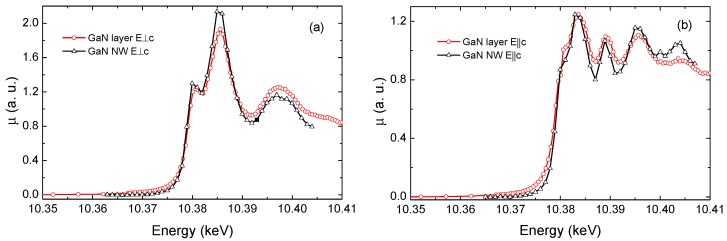
Comparison between X-ray absorption near edge structure (XANES) spectra of a high quality reference GaN layer (red cercles) and those of the NW (black triangles) for different X-ray beam incident angles: polarization (**a**) perpendicular and (**b**) parallel to the wurtzite *c*-axis.

While previous XRD measurements were a probe of the long-range structural order, XANES measurements were performed for studying the local arrangement around Ga atoms in the NWs. The spectra were taken with a step size of 1 eV near the Ga K-edge that matches the energy resolution provided by the Si (111) double-crystal monochromator. A representative XANES of a single NW taken with the polarisation of the X-ray beam perpendicular and parallel to the axis of the NW is shown in Figure 5a,b, respectively. The XANES spectrum of the NW was compared with that measured in a high-quality *c*-oriented GaN reference layer, measured with the polarization of the X-rays either perpendicular or parallel to the wurtzite *c*-axis. The spectra of the NW match the reference ones; therefore, it can be concluded that the NW has wurtzite structure and there is no mixture of phases. Moreover, it can be argued that the axis of the NW coincides with the *c*-axis of the wurtzite structure. Similar results were obtained in the case of parallel polarisation. The small difference between the absorption edge of the NW and that of the layer is attributed to the calibration, since the measurements were carried out in different moments and therefore not exactly under the same experimental conditions.

### 3.4. Emission Properties of Individual NWs

**Figure 6:**
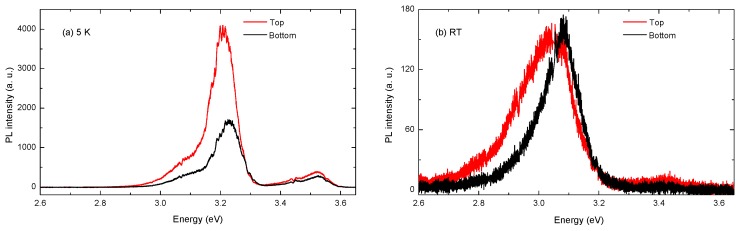
Representative photoluminescence (PL) spectra taken at the top (red line) and at the bottom (black line) of the NWs (**a**) at liquid He and (**b**) room temperature.

The optical properties of individual and ensemble core-shell GaN/InGaN NWs were studied by PL on a statistically relevant number of individual NWs both at room and liquid He temperature. Hundreds of NWs were dispersed on a Au-patterned substrate for measuring their individual emission with the same experimental conditions. Representative PL spectra taken at 5 K with the laser light focused either at the tip or the base ends of the NWs are shown in Figure 6a. All the spectra are dominated by transitions of the GaN/InGaN core-shell MQWs with energies between 3.1–3.3 eV. The large FWHM (~34 meV) of this peak is attributed to the variation of the alloy composition along the NWs. The peak centred around 3.47 eV is attributed to emission from GaN and it is originated by the overlapping of the *D^0^X_A_* (FWHM~50 meV) and *X_A_* emissions (FWHM~20 meV). There is a feature appearing at 3.45 eV present in all the spectra that has been attributed to an artefact coming from the gratings of the spectrometer.

When comparing the spectra taken at the tip and the base of each NW, the PL band of InGaN MQWs increases its intensity and red-shifts from the bottom to the top of the NW. The red-shift follows from the higher In concentration towards the top end of the NW, which was evidenced by the XRF measurements. Despite the inhomogeneities present in each single NW, affecting the In concentration mostly, the PL spectra obtained from individual NWs tend to fit to a single Gaussian peak better than those recorded for NW ensembles (see Figure S4). The MQWs emission identified in macro-PL measurements often presents multi-Gaussian distributions with much greater dispersion than single NW emission. Thus, the overall inhomogeneities of the ensemble mask the real properties of the individual NWs, proving the necessity of single NW characterization.

Figure 6b shows two room temperature spectra of the same individual NW taken at its top and bottom. PL measurements as a function of the temperature allow one to calculate the relative internal quantum efficiency (IQE) of the optical transitions at room temperature. This can be done under the assumption that, at low temperature, non-radiative processes are suppressed. Thus, the relative IQE is given by the ratio between the PL integrated intensity at room temperature and low temperatures. At room temperature, IQE of the tip and of the base of the core-shell InGaN MQWs is around 20% and 15%, respectively. On the other hand, the IQE of the core is less than 1%, as it is expected for the absence of quantum confinement.

### 3.5. Theoretical Simulations

In order to get further insight into the optical properties of the investigated non-polar InGaN/GaN QW NWs, a theoretical model has been implemented. First of all, XRD results point out that strain fields are present in the heterostructures that will affect strongly their optical response. The strain field calculations in 3D systems tend to be time- and CPU-expensive. Thus, it is preferable to reduce the problem to a 2D one by assuming that the strain depends only on the in-plane coordinates (*x*, *y*) and is invariant along *z*. This approximation gives good results in the case of a finite but long 3D system, which perfectly matches the geometry of the NWs studied here [28]. The numerical calculations have been performed using the COMSOL-Multiphysics software platform [29] for a GaN/InGaN MQW NW with averaged values of the thickness of the wells and the barriers (4.5 and 5.4 nm, respectively, resulting from EDS measurements) and In concentration of 10% (extracted from XRF data). It is assumed that the only source of strain is given by the lattice-mismatch between InGaN and GaN.

**Figure 7:**
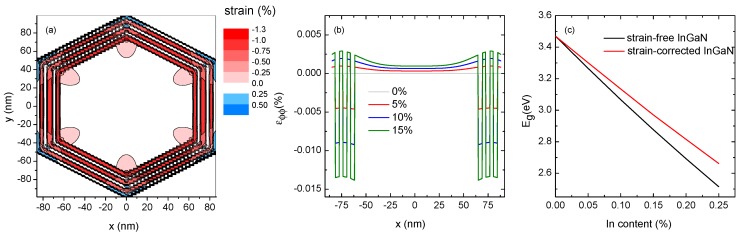
(**a**) Cross-sectional map of the angular strain component (ε) for InGaN MQWs with 10% In concentration. (**b**) Evolution of the angular strain component along the *x*-direction (*y* = 0) for different In concentrations. (**c**) Band gap energy of strain-free and strained InGaN for different In concentrations.

A cross-sectional contour map of the angular strain component (ε in the chosen cylindrical coordinates) is represented in Figure 7a. The GaN core is essentially relaxed (all strain components are in the range 0–0.2%). Inside the InGaN QWs, ε_ϕϕ_~−0.8% (similar values are obtained for ε_zz_, see Figure S5) and indicates that the three QWs are under compressive strain due to the larger lattice parameter of InGaN compared to that of GaN. The radial component is tensile strained as a result of the compression along the angular and *c*-axis directions (ε_rr_~0.4\%). The distribution of the strain is homogeneous, except for the corners where, due to their particular shape, the strain field has a more complicated behavior. These results confirm the observations made by the XRD: GaN/InGaN MQWs grow strained and matched to the GaN material. Figure 7b presents the evolution of the angular strain component along the diameter of the NW.

The effect of changing the In concentration has been modeled from 0 up to 15% and, within this range, the average value of the strain components changes linearly. Using the obtained strain field distribution, the electronic band structure of strained InGaN can be calculated. Figure 7c shows the trend of InGaN bandgap energy (E_g_) at low temperatures for different In concentrations. The strain-free values of the InGaN bandgap are also plotted for comparison. As expected, due to the compressive character of the strain field, strained InGaN has band gap energies higher than those of relaxed material. The values of In concentration in the GaN/InGaN core-shell MQWs, obtained from the XRF maps, are in the range of 5–12%, and the corresponding bandgap energies are in the 3.05–3.29 eV range. This result is in very good agreement with the PL measurements. The large FWHM (~34 meV) of the emission is also consistent with the variation of In concentration along the NW axis.

## 4. Conclusions

The elemental, structural, and optical characterization of GaN/InGaN core-shell NWs requires a battery of complementary and non-destructive techniques. Due to the complexity of these nanostructures, including inhomogeneities and strain at a nanometer length scale, the combination of more than one technique, in such a manner that the strengths of one complement the weaknesses of others, is indispensable. The combination of XRF and EDS gives a reliable picture of the dimensions of the QWs and their alloy composition. Variations in In content along the axial direction are present in all NWs, and the upper tips tend to be In richer than the bottom ends. XANES and XRD probe local and long-range order in the heterostructures. Knowing the In concentration in the QWs, their strain state can be inferred from XRD spectra. Finally, photoluminescence spectra of individual NWs can be interpreted and modeled theoretically. Fluctuations in the In content can explain the variations in the emission energy and the broadening of the emission peaks.

## Figures and Tables

**Table 1:** Values of the In concentration of the core-shell multiple quantum wells (MQWs) at the different positions along the axis of the NW indicated in Figure 3c.

Point	*C_In_* (%)
A	11.6 ± 3.3
B	9.2 ± 2.7
C	7.4 ± 2.1
D	6.3 ± 1.8
E	5.4 ± 1.6
F	5.4 ± 1.6

## Supplementary Materials

The following are available online at https://www.mdpi.com/2079-4991/9/5/691/s1, Figure S1: EDS linear scan along the NW axis, Figure S2: XRF maps of Au-L and Ag-Kα, Figure S3: Linear scans of In and Ga XRF peaks performed along the NW axis and NW diameter, Figure S4: PL spectrum of an ensemble of NWs, Figure S5: Contour plots of the strain components on the NW cross-section.
