# Supplementary material for: Elemental Distribution and Structural Characterization of GaN/InGaN Core-Shell Single Nanowires by Hard X-ray Synchrotron Nanoprobes
Source: Nanomaterials (Basel). 2019 May 3;9(5):691. doi: 10.3390/nano9050691 (PMC6566811; doi:10.3390/nano9050691)
Supplement: Supplementary file 1 [file nanomaterials-09-00691-s001.pdf]

*nanomaterials*

*Supplementary*

# Elemental Distribution and Structural Characterization of GaN/InGaN Core-Shell Single Nanowires by Hard X-ray Synchrotron Nanoprobes

**Eleonora Secco [1], Heruy Taddese Mengistu [1], Jaime Segura-Ruíz [2], Gema Martínez-Criado [2,3], Alberto García-Cristóbal [1], Andrés Cantarero [4], Bartosz Foltynski [5], Hannes Behmenburg [5], Christoph Giesen [5], Michael Heuken [5] and Núria Garro [1,*]**

1 Institut de Ciència dels Materials (ICMUV), Universitat de València, 46980 Paterna (València), Spain; eleonora.secco@uv.es (E.S.); Heruy.Mengistu@uv.es (H.T.M.); alberto.garcia@uv.es (A.G.-C.)
2 ESRF – The European Synchrotron, 71 avenue des Martyrs, 38043 Grenoble, France; jaime.segura@esrf.fr (J.S.-R.); gema.martinez.criado@csic.es (G.M.-C.)
3 Instituto de Ciencia de Materiales de Madrid (ICMM), Consejo Superior de Investigaciones Científicas (CSIC), Sor Juana Inés de la Cruz 3, 28049 Madrid, Spain
4 Institut de Ciència Maolecular (ICMOL), Universitat de València, 46980 Paterna (València), Spain; andres.cantarero@uv.es
5 AIXTRON SE, Dornkaulstrasse 2 52134 Herzogenrath, Germany; B.Foltynkski@aixtron.com (B.F.); H.Behmenburg@aixtron.com (H.B.); C.Giesen@aixtron.com (C.G.); M.Heuken@aixtron.com (M.H.)
* Correspondence: nuria.garro@uv.es; Tel.: +34-9635-436-01

Core-shell MQWs GaN/InGaN on GaN NWs were grown by MOCVD in an AIXTRON 3 × 2″ close-coupled showerhead reactor. The standard precursors were triethylgallium (TEGa) and trimethylindium (TMIn) for the metal species and ammonia ($NH_3$) for the nitrogen supply. $N_2$ was used as the carrier gas. The VLS Au catalyst initiated growth of GaN NWs was realised by utilising the sapphire substrate coated ex-situ with an Au film of 1 nm of nominal thickness. At the beginning of the growth process, after reaching the growth temperature of around 1020 °C, TEGa supply was applied for the first two minutes to allow Ga enrichment of the Au catalyst. This pre-deposition step was followed by the simultaneous introduction of both TEGa and ammonia supply. The V/III ratio was of around 3 and promoted the vertical growth of the NWs. Three layers of InGaN were deposited at a lower temperature of 730 °C and followed by three GaN layers.

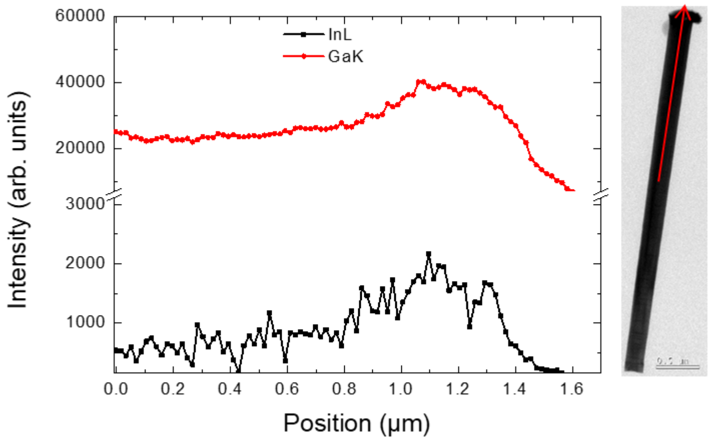


**Figure S1.** EDS integrated intensities linear profiles of the L$\alpha$-line of In and the K$\alpha$-line of Ga performed along the axial direction of the NW, as depicted in the HRTEM image.

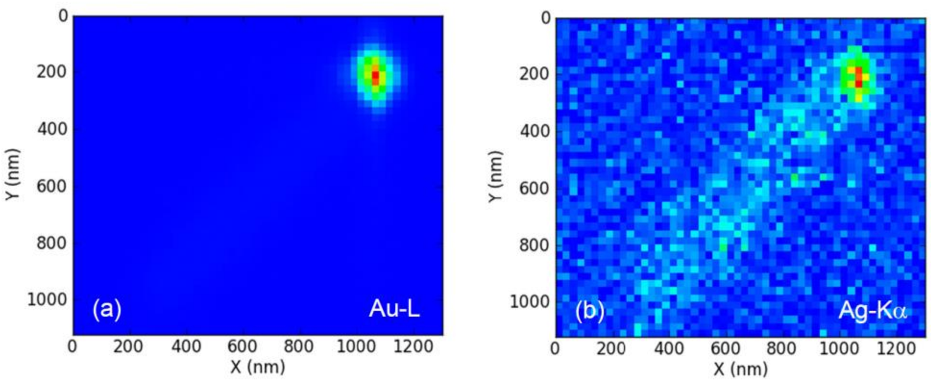


**Figure S2.** XRF colour map distribution of (**a**) Au and (**b**) Ag along a representative NW. The colour scale represents the XRF intensity in photon counts and ranges from 0 to 9000 (for Au) and from 0 to 25 (for Ag).

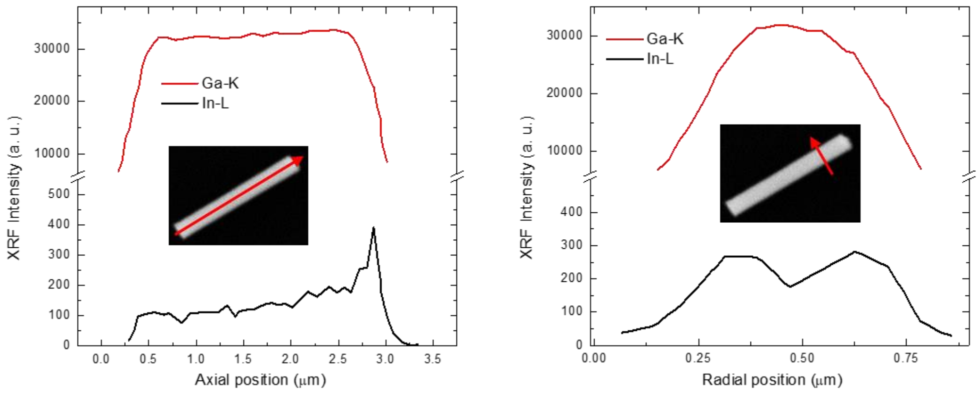


**Figure S3.** Integrated intensities linear profiles of In and Ga XRF peaks performed along the NW axis (left hand side figure) and NW diameter (right hand side figure).

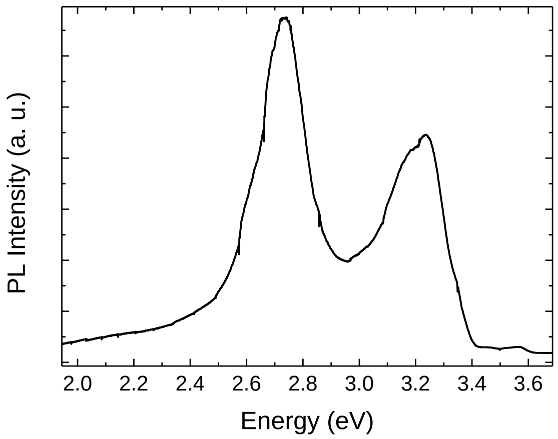


**Figure S4.** Macro-PL spectrum corresponding to an ensemble of GaN/InGaN NWs measured at liquid He temperature.

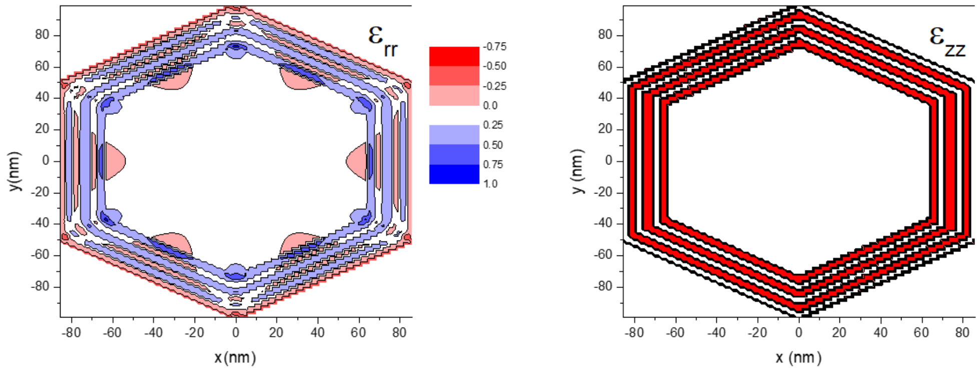


**Figure S5.** Strain field maps of the NW cross-section for InGaN MQWs with 10\% In concentration for strain components along radial ($\varepsilon_{rr}$) and axial ($\varepsilon_{zz}$) directions.

**Supplementary Materials:** The following are available online at www.mdpi.com/xxx/s1, detailed description of the NW growth, Figure S1: EDS linear scan along the NW axis, Figure S2: XRF maps of Au-L and Ag-K$\alpha$, Figure S3: Linear scans of In and Ga XRF peaks performed along the NW axis and NW diameter, Figure S4: PL spectrum of an ensemble of NWs, Figure S5: Contour plots of the strain components on the NW cross-section

© 2019 by the authors. Submitted for possible open access publication under the terms and conditions of the Creative Commons Attribution (CC BY) license (http://creativecommons.org/licenses/by/4.0/).
